# Depression, Insomnia and Post-Traumatic Stress Disorder in COVID-19 Survivors: Role of Gender and Impact on Quality of Life

**DOI:** 10.3390/jpm12030486

**Published:** 2022-03-17

**Authors:** Sofia Pappa, Zafeiria Barmparessou, Nikolaos Athanasiou, Elpitha Sakka, Kostas Eleftheriou, Stavros Patrinos, Nikolaos Sakkas, Apostolis Pappas, Ioannis Kalomenidis, Paraskevi Katsaounou

**Affiliations:** 1 Department of Brain Sciences, Imperial College London, London W12 0NN, UK; 2 Pulmonary and Respiratory Failure Department, First ICU, Evangelismos Hospital, 106 76 Athens, Greece; zafeiria.barmparessou@gmail.com (Z.B.); nikolaosathanasiou14@gmail.com (N.A.); coselef@hotmail.com (K.E.); stavrosspatrinos@gmail.com (S.P.); apo.pappas88@gmail.com (A.P.); ikalom@med.uoa.gr (I.K.); paraskevikatsaounou@gmail.com (P.K.); 3 School of Pharmacy and Biomolecular Sciences, University of Brighton, Brighton BN2 4AT, UK; elpithasakka@hotmail.com; 4 Faculty of Medicine, University of Warwick, Coventry CV4 7AL, UK; nicksakkas1@gmail.com; 5 Medical School, National and Kapodistrian University of Athens, 157 72 Athens, Greece

**Keywords:** COVID-19, patients, mental health, depression, anxiety, PTSD, sleep, quality of life, sex differences

## Abstract

Evidence to date suggests that a significant proportion of COVID-19 patients experience adverse psychological outcomes and neuropsychiatric complications. The aim of this study was to evaluate the effect of SARS-CoV-2 infection and subsequent hospitalization on the mental health, sleep, and quality of life of COVID-19 survivors. Patients were assessed 1–2 months after hospital discharge using standardized screening tools for depression and anxiety (HADS), post-traumatic stress disorder (IES-R), insomnia (AIS), and quality of life (EQ-5D-5L). Sociodemographic factors, comorbidities, disease severity and type of hospitalization were also collected. Amongst the 143 patients included, mental health symptoms were common (depression—19%; anxiety—27%; traumatic stress—39%; insomnia—33%) and more frequently reported in female than in male patients. Age, smoking status, comorbidities and illness severity were not found to significantly correlate with the presence of mood, sleep, or stress disorders. Finally, quality of life was worse for patients requiring ICU (*p* = 0.0057) or a longer hospital stay (*p* < 0.001) but was unaffected by factors such as sex and other measured outcomes. These findings highlight the need for appropriate intervention to properly manage the immediate and enduring mental health complications of COVID-19.

## 1. Introduction

A severe acute respiratory syndrome caused by a novel coronavirus (SARS-CoV-2) was identified in Wuhan, China in December 2019 and was later named Coronavirus Disease 2019 (COVID-19) [1,2]. COVID-19 was declared a pandemic by the World Health Organization (WHO) on 11 March 2020 [1] and has since led to a large-scale global health crisis. SARS-CoV-1 and MERS-CoV are notable homologous viruses with SARS-CoV-2, which were responsible for causing the SARS epidemic and MERS outbreak in 2003 and 2012, respectively [3].

These and other infectious disease outbreaks such as Ebola in 2013 were shown to affect the mental health of the general population and high-risk groups [4,5,6,7] such as patients. In fact, evidence relating to the previous coronaviruses demonstrated that infected patients were at an increased risk of experiencing symptoms of psychological distress and developing mental health disorders, including depression, anxiety, post-traumatic stress disorder, and sleep disturbances [8].

Although a large proportion of early research following COVID-19 focused on the physiological effects of the virus, a substantial body of subsequent studies have shown that the psychological burden of COVID-19 infection is considerable and that COVID-19 patients may experience an excess of adverse psychological outcomes and neuropsychiatric complications [1,4,7,8]. Thus, alongside a number of other psychosocial factors, a COVID-19 diagnosis is more likely to increase susceptibility to developing mood, cognitive, and sleep disturbances as infected patients often require hospitalization in the ICU (Intensive Care Unit) and mechanical ventilation, both of which are considered risk factors for the acquisition of acute psychiatric disorders; direct bio-immunological effects have also been implicated [9,10].

Prevalence rates vary considerably across studies and the literature is still maturing on the topic, but there is increasing evidence showing that psychological and neuropsychiatric symptoms tend to persist after active infection and/or post-hospitalization [11]. In fact, a large retrospective cohort study that used electronic health records of 236,379 patients and was published in the Lancet, by Taquet et al. [12], found that the incidence of neurological or psychiatric diagnoses at 6 months post-COVID-19 infection was approximately 33%, of which 13% received a first diagnosis in this period of time. Furthermore, these were more common in patients with COVID-19 compared to those who had influenza or other respiratory infections [12].

A systematic review and meta-analysis, including data from 47,910 patients, showed that up to 80% of infected patients developed at least one long-lasting symptom, including fatigue (58%), memory loss (16%), anxiety (13%) and depression (12%) [13]. Another recently published systematic review including 51 studies (n = 18,917 patients) also reported persistent problems with sleep disturbance (27%), anxiety (19%), depression PTSD (16%), and cognitive impairment (20%). There was little or no evidence of differences in prevalence based on hospitalization status, disease severity or follow-up duration [14].

However, the consequences on the mental health and quality of life of COVID-19 patients that required hospitalization remain largely understudied. Therefore, the aim of this study was to investigate the impact of COVID-19 infection and hospitalization on the mental health, quality of life, and sleep of patients following hospital discharge. Early identification and timely management of these disorders is paramount in improving prognosis, reducing duration of hospitalization, and preventing the development of long-term mental health issues in patients suffering from COVID-19.

## 2. Materials and Methods

### 2.1. Study Design and Population

We performed a cross-sectional study at a large COVID-19 tertiary reference center (Evangelismos Hospital) in Athens, Greece. Patients surviving COVID-19 who had been admitted for their symptomatology with PCR-confirmed SARS-CoV-2 infection from 8 May 2020 until August 2021 were assessed in the outpatient clinic 1–2 months after hospital discharge. Participants completed a questionnaire on their first appointment, following informed consent, and they were allowed to terminate the survey at any time if they wished to. Patients who were younger than 18, lacked capacity or had language barriers, were excluded from the study. The study obtained approval from the clinical research ethics committee of Evangelismos General Hospital (Ethical Approval Number—AΠ 173/24-4-2020).

### 2.2. Questionnaire and Psychometric Scales

The self-administered questionnaire included sociodemographic information, smoking history, comorbidities, and psychometric scales assessing levels of fear, anxiety, depression, insomnia, traumatic stress, and quality of life. Disease severity, duration, and type of hospitalization due to SARS-CoV-2 infection were collected from the patients’ records.

The questionnaire included:

(1) Sociodemographic and clinical factors: gender, age, body mass index (BMI), smoking, comorbidities, disease severity, hospitalization in COVID-19 clinic or ICU, and days of hospitalization (total and ICU).
  Disease severity was assessed according to the National Institute of Health criteria [15] as follows:
  - Mild Illness: Individuals who have any of the various signs and symptoms of COVID-19 (e.g., fever, cough, sore throat, malaise, headache, muscle pain, nausea, vomiting, diarrhea, loss of taste and smell) but who do not have shortness of breath, dyspnea, or abnormal chest imaging.
  - Moderate Illness: Individuals who show evidence of lower respiratory disease during clinical assessment or imaging and who have an oxygen saturation (SpO_2_) ≥ 94% on room air at sea level.
  - Severe Illness: Individuals who have SpO_2_ < 94% on room air at sea level, a ratio of arterial partial pressure of oxygen to fraction of inspired oxygen (PaO_2_/FiO_2_) < 300 mm Hg, a respiratory rate > 30 breaths/min, or lung infiltrates > 50%.
  - Critical Illness: Individuals who have respiratory failure, septic shock, and/or multiple organ dysfunction.

Critically ill patients as well as patients with severe infection and comorbidities were transferred to ICU for better monitoring.

(2) Psychometric Scales [16,17,18,19,20,21,22]:
  - The Hospital Anxiety and Depression Scale (HADS) is a 14-item self-administered screening tool for the presence of depression and anxiety [16]. Respondents are asked to reflect on their mood in the past week. On the scale, 7 items assess depression and 7 items assess anxiety. Scores for items in each subscale of the HADS are summed to produce an anxiety score (HADS-A) or a Depression score (HADS-D) or can be added to produce a total score (HADS-T). Total scores range between 0 and 21 for each scale and are graded for severity from normal (0–7), mild (8–10), moderate (11–14) to severe (15–21). The scale has been validated in Greek, demonstrating good psychometric properties, i.e., high internal consistency (Cronbach’s alpha coefficient was 0.884) and stability (test–retest correlation coefficient 0.944) while factor analysis confirmed a two-factor structure [16].
  - Impact of Event Scale-Revised (IES-R) is a validated 22-item self-report that measures subjective psychological distress in response to traumatic events [17,18]. It has 3 subscales (Intrusion, Avoidance and Hyperarousal), which are closely associated with post-traumatic stress disorder (PTSD) symptoms. Total scores range between 0 and 88 and are graded for severity from normal (0–23), mild (24–32), moderate (33–36) to severe psychological distress (>37). A cut-off score of 24 is commonly used to define PTSD of a clinical concern. The Greek version used has shown good psychometric features; the Cronbach’s alphas for the intrusion, avoidance, and hyperarousal scales were 0.72, 0.77, and 0.85, respectively; overall test–retest reliability was also satisfactory [17].
  - The AIS is a questionnaire developed to evaluate insomnia problems [19]. Each item is rated from 0 (no problem at all), 1 (mild problem), and 2 (marked problem), to 3 (very serious problem). The first 5 items assess difficulty with sleep induction, awakenings during the night, early morning awakening, total sleep time, and overall sleep quality. The last three items assess the next-day consequences of insomnia, such as problems with sense of wellbeing, functioning, and daytime sleepiness. A cut-off score of >6 is used to establish the diagnosis of insomnia. The Greek version was used; the scale has shown very good psychometric characteristics (with Cronbach’s α around 0.90 and test–retest reliability correlation coefficient at almost 0.90) [19].
  - Numerical fear rating scale (NFRS) was used to measure the level of fear in the study, which has been reported to have good reliability and validity [20]. It is a segmented numeric version of the visual analog scale (VAS) in which a respondent selects a whole number (0–10 integers) that best reflects the intensity of their fear. Higher scores indicate greater fear as follows: 0 for no fear, 1–3 for mild fear, 4–6 for moderate fear, 7–9 for severe fear, 10 for extreme fear.
  - Quality of Life (EQ-5D-5L) essentially consists of 2 parts: the EQ-5D descriptive system and the EQ visual analogue scale (EQ-5D-5L VAS) [21,22], which we report on here. The descriptive system consists of 5 dimensions: mobility, self-care, usual activities, pain/discomfort and anxiety/depression. The EQ-5D-5L VAS records the patient’s self-rated health on a vertical visual analogue scale from the best to worst health and can be used as a patient-reported quantitative measure of health outcome. The scale has been validated in Greek, showing good performance in terms of low ceiling effects, high absolute and relative informativity, and convergent and known-group validity efficiency [21].

### 2.3. Statistical Analysis

Descriptive statistics were used for the sociodemographic information and other continuous outcome variables including, fear, anxiety, depression, traumatic stress and insomnia; categorical variables were expressed as percentages and continuous variables as mean values ± (standard deviation). Welch’s *t*-test and Multivariate Analysis of Variance (MANOVA) were used to examine the association between continuous variables. Two-tailed *p* values of less than 0.05 were deemed statistically significant.

## 3. Results

### 3.1. Demographic and Participant Characteristics

**Figure 1:**
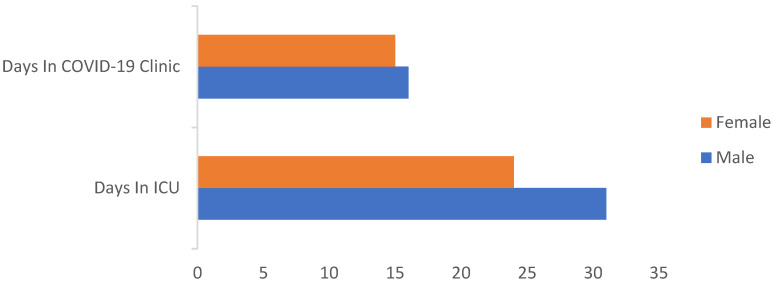
Length and type of hospitalization by gender.

A total of 143 patients with a mean age of 57.1 (SD: 11) that were assessed in the outpatient clinic following hospitalization for COVID-19 infection participated in the study. The demographic and clinical characteristics of the study population are summarized in Table 1. It is also noteworthy that most patients required long hospitalizations due to the severity of their illness, and that 18% had to be admitted to an ICU; furthermore, female patients had shorter duration of hospitalizations (14.9 vs. 18.8 bed days) and/or ICU admissions (13.5 vs. 14.9), though this did not prove to be statistically significant (Figure 1).

### 3.2. Psychometric Scales Outcomes

**Figure 2:**
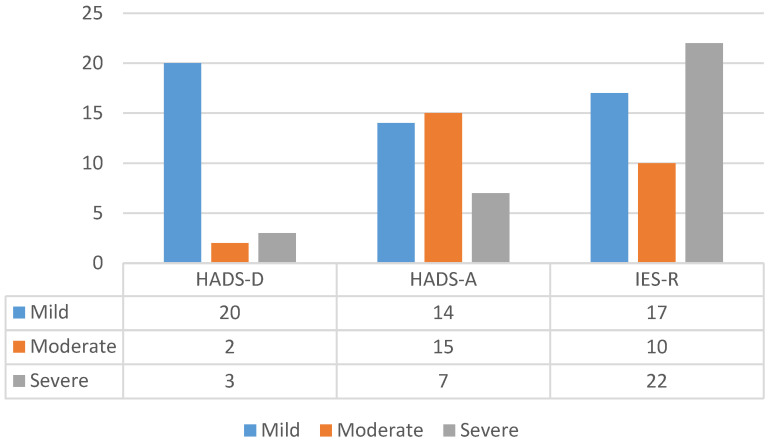
Number of COVID-19 patients with mild, moderate, and severe symptoms of depression, anxiety, and traumatic stress. HADS-D = Hospital Anxiety and Depression Scale—Depression; HADS-A = Hospital Anxiety and Depression Scale—Anxiety; IES-R = Impact of Event Scale—Revised.

**Figure 3:**
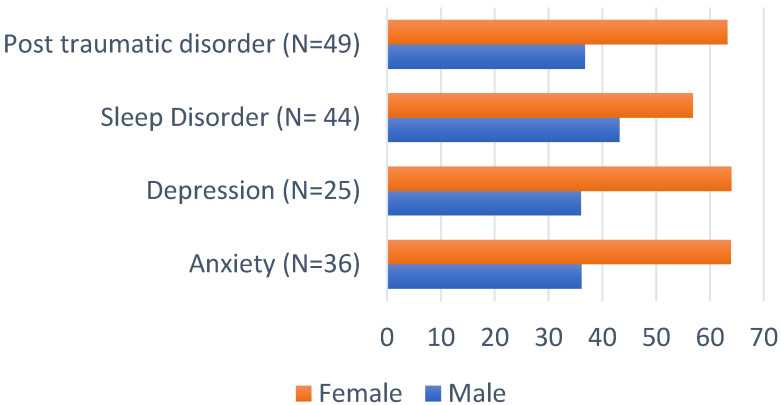
Sex differences in depression, anxiety, insomnia, and PTSD.

A total of 133 patients completed all the psychometric scales and were included in the final analysis. COVID-19 patients experienced high levels of depression, anxiety, traumatic stress, and sleep disorders (Table 2, and Figure 2 and Figure 3). As shown, rates of depression, anxiety, stress, fear, and sleep problems were all significantly higher in female compared to male patients. Age, smoking, comorbidities, and severity of illness were not found to significantly correlate with the presence of depression, anxiety, stress, fear or insomnia.

As illustrated in Table 3, quality of life was worse for patients that have had an admission to the ICU (EQ-5D-5L VAS): 49.7 ± 19.3) compared to those who were hospitalized but did not require ICU treatment (EQ-5D-5LVAS): 65.7 ± 21.2) (*p* = 0.0057) and for those requiring longer hospitalizations (*p* < 0.001). Scores were not significantly associated with other factors such as sex, comorbidity, depression, anxiety, sleep dysfunction, level of fear or stress.

## 4. Discussion

The study findings detail the residual mental health effects of a SARS-CoV-2 infection on affected patients, with mood, sleep, trauma, and stress-related symptoms persisting for months post-disease onset and hospital discharge. Overall, these appear considerable, particularly for sleep difficulties and PTSD-type symptoms, and comparable with previously reported estimates of similar studies [23] and the mental health effects reported in frontline HCWs in Greece during the first wave of the pandemic [24]. However, it is important to note that there is significant variability in reported figures across all psychological outcomes in different studies. Confounding factors include, amongst others, differences in study populations and the application of different assessment scales and cut-off scores, thus introducing great between-study heterogeneity [24,25].

The most consistent study finding was that of significantly higher prevalence rates of adverse mental health symptoms in female compared to male patients despite displaying significantly lower duration of hospitalizations and frequency of ICU admissions. This observation is in line with a previous study, showing that, despite significantly lower levels of baseline inflammatory markers, female patients suffered more in terms of both anxiety and depression at one-month follow-up following hospital admission [10]. In fact, sex differences were consistent across all recorded psychological outcomes, i.e., depression (*p* = 0.005), anxiety (*p* < 0.001), traumatic stress (*p* < 0.001), fear (*p* < 0.001) and insomnia (*p* < 0.001), while being particularly marked for moderate levels of depression and traumatic stress and for severe levels of anxiety.

It is well known that, overall, females are more likely to suffer from depression and anxiety and are at a higher risk of developing PTSD following exposure to traumatic events compared to males [26]. A previous longitudinal study revealed that female gender and chronic comorbidities were independent predictors of the subsequent emergence of PTSD in SARS survivors [27]. Akin to our own results, a number of other studies to date confirmed the higher likelihood of female COVID-19 patients and survivors to experience mental health problems compared to their male counterparts [28].

Apart from gender, common risk factors identified previously include disease severity, length of stay and presence of comorbidity [23,29,30]. In our study, factors such as age, smoking, comorbidities, and severity were not found to significantly correlate with the presence of mood symptoms or sleep dysfunction, which is not dissimilar to a number of other studies that failed to detect any significant associations of COVID-19 psychological sequelae, particularly with the survivors’ age.

Unsurprisingly, quality of life was significantly worse for patients that have had an admission to the ICU and a longer stay in hospital but was unrelated to sex, the presence of comorbidities or any of the mental health outcomes. In the systematic review by Dorri et al. [28], COVID-19 survivors showed reduced HRQoL and a lower score in Social Functioning (SF) compared to pre-COVID-19 and controls, though risk factors such as female gender, adverse psychological outcomes and ICU admission varied across included studies.

### 4.1. Depression and Anxiety

Depressive and anxious symptoms were the least prevalently reported in this cohort, representing 18.8% and 27.1% of COVID-19 survivors 1–2 months post-discharge. Overall, 18.8% of participants reported at least mild depressive symptoms but of these, only 3.8% were moderate to severe. Anxiety symptoms were more common in comparison to those of depression, which is consistent with most studies to date [31], and of the 27.1% of the recovered participants that experienced at least mild feelings of anxiety, 16.5% reported moderate to severe symptoms.

Rates of depression and anxiety in COVID-19 patients were found to be 34% and 47% in the acute phase of the illness, as demonstrated in a recent systematic review and meta-analysis by Deng et al. [8] but substantially lower 6 months post-discharge, as demonstrated in a rapid study in China by Huang et al. [32], with 23% of the 1733 patients experiencing symptoms of depression and anxiety. This decline in anxious and depressive feelings may well result from the relief of recovery and hospital discharge. In fact, in a systematic review of 21 studies [28], the pooled prevalence of depression and anxiety among COVID-19 survivors was 12% (8 to 17%) and 17% (12 to 22%), respectively. Similarly, a recent meta-analysis revealed that PTSD (32.2%), anxiety disorders (14.8%), and depression (14.9%) were prevalent among survivors of other coronaviruses at a mean follow up of 35 months [7].

### 4.2. Traumatic Stress and PTSD

A considerable proportion of discharged COVID-19 patients experienced symptoms of traumatic stress and PTSD, with 37.4% reporting at least mild symptoms of stress; of these, the majority (24.4%) reported moderate to severe symptoms.

The prevalence of traumatic stress demonstrated in this study appears to be of a similar range to those found in other studies to date. For example, a U.K. study by Halpin et al. [33] found that 47% of their hospitalized cohort (of 68 participants) reported symptoms of traumatic stress related to their COVID-19 diagnosis 1–2 months after discharge. Additionally, ICU patients (*n* = 32) appeared to suffer from symptoms of PTSD and traumatic stress at higher levels than other ward patients in this U.K. cohort, with 46.9% presenting with at least mild symptoms of PTSD [33]. In an earlier study of 115 discharged patients in Italy, 10.4% of the sample received a PTSD diagnosis, while a further 8.6% met three out of four of the criteria at 3 months post-discharge [31]. In the aforementioned systematic review by Dorri et al. [28], the pooled prevalence of PTSD among COVID-19 survivors was 18%; patients with severe disease displayed higher prevalence of depression and anxiety, but not PTSD.

### 4.3. Insomnia

One-third (33%) of included participants experienced poor-quality sleep and insomnia, evaluated using the AIS. Prevalence rates of insomnia amongst discharged COVID-19 patients in this study appear to be towards the higher end than rates reported previously. A recent systematic review of 10 studies assessing sleep quality, utilizing the PSQI (Pittsburgh Sleep Quality Index) questionnaire, found that approximately one in four COVID-19 survivors were diagnosed with a sleep disorder (with prevalence rates ranging from 19.2 to 30.3%) [34]. Similar rates were reported in another systematic review, which included 52 studies and 18,917 participants; in fact, disturbed sleep was the most frequently reported neuropsychiatric symptom in participants 14–182 days after recovery from COVID-19, with a pooled prevalence of 27.4% [35].

Likewise, an early in-person follow-up study in Wuhan, China, found sleep abnormalities to be one of the most reported complications amongst 1733 discharged patients 6 months post-disease onset, affecting roughly 26% of their cohort [29]. Similar findings were reported in studies across Europe, including France and the U.K. where sleep difficulties affected 30.8% and 24% of survivors, respectively, 3–4 months following symptom onset and hospitalization [14,36].

## 5. Conclusions

COVID-19 disease was found to have a considerable psychological impact on hospitalized patients post-discharge. Furthermore, rates of depression, anxiety, traumatic stress, and insomnia were significantly worse for female patients despite the less severe course of their illness (duration and ICU). Unlike sex, age, smoking status and co-morbidities did not show a significant correlation with the presence of mood, stress or sleep disorders, while poorer quality of life was associated with ICU admission. Our results highlight the need for appropriate interventions to promote the physical and mental wellbeing of COVID-19 survivors and cater for long-term needs.

## Figures and Tables

**Table 1:** Sample characteristics.

Age	N	Mean ± SD
Male/Female	143	57.10 ± 13
**Gender**	**N**	**%**
Male	91	63.64
Female	52	36.36
**Severity Of COVID-19**		
Mild	4	2.80
Moderate	50	34.97
Severe	69	48.25
Critical	20	13.99
**Comorbidities**	108 (with a total of 161 comorbidities)	75.52
Diabetes mellitus	12	8.39
Hypertension	44	30.99
Coronary disease	6	4.20
Cancer	10	6.99
Immunosuppression	11	7.69
Asthma	20	13.99
COPD	5	3.50
Obesity	53	38.69
**Smoking Status**		
Never smoker	80	56.74
Ex-smoker	44	31.21
Current Smoker	17	12.06
Not known	2	
**Hospitalization**		
COVID-19 Clinic	118	81.88
Intensive Care Unit	25	18.12

**Table 2:** Psychometric scales outcomes: levels of severity. (*: *p* < 0.05; **: *p* < 0.01; ***: *p* < 0.001).

	Male	Female	Total	
HADS Depression	N (%)	N (%)	N (%)	*p*-Value
No depression	75 (69.44)	33 (30.56)	108 (81.2)	
Mild	8 (40.00)	12 (60.00)	20 (15.04)	=0.005 **
Moderate	0 (0.00)	2 (100.00)	2 (1.05)	
Severe	1 (33.33)	2 (66.67)	3 (2.26)	
**HADS Anxiety**				
No Anxiety	71 (73.20)	26 (26.80)	97 (73.93)	
Mild	4 (28.57)	10 (71.43)	14 (10.53)	<0.001 ***
Moderate	7 (46.67)	8 (53.33)	15 (11.28)	
Severe	2 (28.57)	5 (71.43)	7 (5.26)	
**AIS**				
No insomnia	64 (72.73)	24 (27.27)	88 (66.67)	<0.001 ***
Insomnia	19 (43.18)	25 (56.82)	44 (33.33)	
**IES-R**				
No stress	65 (79.27)	17 (20.73)	82 (62.6)	
Mild	8 (47.06)	9 (52.94)	17 (12.98)	<0.001 ***
Moderate	1 (10.00)	9 (90.00)	10 (7.63)	
Severe	10 (45.45)	12 (54.55)	22 (16.79)	
**Severity of COVID-19 Illness**				
Mild	1 (25.00)	3 (75.00)	4 (2.7)	
Moderate	25 (50.00)	25 (50.00)	50 (34.96)	=0.007 **
Severe	48 (69.57)	21 (30.43)	69 (48.25)	
Critical	17 (85.00)	3 (15.00)	20 (13.98)	
	**Mean ± SD**	**Mean ± SD**	**(Mean ± SD)**	
**Fear**	3.16 ± 2.64	5 (5.59 ± 2.91)	(4.06 ± 2.98)	<0.001 ***

**Table 3:** Quality of life: EQ-5D-5L VAS scores. (*: *p* < 0.05; **: *p* < 0.01; ***: *p* < 0.001).

EQ-5D-5L (VAS)	Median	Mean ± SD	*p*-Value
HADS			
No depression	65	64.5 ± 19.6	0.05
Mild	70	59.6 ± 25.9	
Moderate	--	--	
Severe depression	30	30 ± 14.1	
No anxiety	70	65.9 ± 18.7	0.11
Mild	52.5	52.5 ± 17.7	
Moderate	50	52.8 ± 27.1	
Severe anxiety	45	58.3 ± 33.7	
**AIS**			0.12
No insomnia	70	65.4 ± 19.6	
Insomnia	55	58.2 ± 23.1	
**IES-R**			0.47
No stress	65	64.3 ± 19.4	
Mild stress	65	65 ± 18.9	
Moderate stress	70	62 ± 29.5	
Severe stress	47.5	54.2 ± 27.6	
**Smoking**			
Non-smoker	65	61 ± 21.5	0.68
Ex-smoker	60	63.7 ± 23.5	
Smoker	70	66.4 ± 13.6	
No comorbidities	62.5	61.4 ± 20.4	0.76
Comorbidities	62.5	62.8 ± 21.9	
No ICU admission	70	65.7 ± 21.2	0.005 **
ICU admission	50	49.7 ± 19.3	
Females	60	60.4 ± 22.2	0.48
Males	65	63.5 ± 21.21	

## Data Availability

The data presented in this study are available on request from the corresponding author.
